# Being a Psychotherapist in Times of the Novel Coronavirus Disease: Stress-Level, Job Anxiety, and Fear of Coronavirus Disease Infection in More Than 1,500 Psychotherapists in Austria

**DOI:** 10.3389/fpsyg.2020.559100

**Published:** 2020-09-29

**Authors:** Thomas Probst, Elke Humer, Peter Stippl, Christoph Pieh

**Affiliations:** ^1^Department for Psychotherapy and Biopsychosocial Health, Danube University Krems, Krems, Austria; ^2^Austrian Federal Association for Psychotherapy, Vienna, Austria

**Keywords:** psychotherapists, stress, anxiety, fear of infection, coronavirus disease

## Abstract

This study investigated stress-level, degree of job-related anxiety, and fear of coronavirus disease (COVID-19) infection in psychotherapists in the early weeks of the COVID-19 lockdown in Austria. One thousand five hundred and forty-seven psychotherapists participated in an online survey, assessing stress [Perceived Stress Scale-10 (PSS-10)], work-related worries and fears of existence [Job Anxiety Scale (JAS)], fear of COVID-19 infection during face-to-face psychotherapy, and adherence to five protective measures against COVID-19 infection during face-to-face psychotherapy. Stress-levels were higher than in a representative sample (*p* < 0.001). When psychotherapy was the sole income, stress-level (*p* = 0.020) and job anxiety (*p* < 0.001) were higher. Experiences with teletherapy, the psychotherapy format used during COVID-19, as well as reductions in number of patients treated during COVID-19, had no effect on stress-level or job anxiety. Psychotherapists still conducting face-to-face psychotherapy during COVID-19 reported less fear of infection compared to those conducting no face-to-face psychotherapy (*p* < 0.001), whereby the fear of infection was further reduced when they were more able to adhere to protective measures against COVID-19 (*p* < 0.01). Mental hygiene is important for psychotherapists to manage stress and job-related anxiety during COVID-19, especially in those whose income relies on psychotherapy.

## Introduction

Previous research suggested that emotional stressors and existential stressors due to financial concerns range among the major stressors’ psychotherapists are exposed to (Petrowski et al., 2014; Puig et al., 2014). The novel coronavirus disease (COVID-19) and the measures necessary to fight it (i.e., quarantine, isolation, and social distancing; see Nussbaumer-Streit et al., 2020) are new stressors, which can increase and even cause public mental health problems (Brooks et al., 2020; Hossain et al., 2020; Sharma et al., 2020). Mental health care is, therefore, essential during and after COVID-19 (Fiorillo and Gorwood, 2020; Pfefferbaum and North, 2020; Xiang et al., 2020). Psychotherapists are specifically qualified to provide mental health care. Yet, they might face problems in dealing with the impact of COVID-19 on their life and professional activity (Pfefferbaum and North, 2020). For example, sessions are usually provided in personal contact (face-to-face), which has to be reduced now and most likely in the near future as well. Although providing psychotherapy *via* telephone or internet (teletherapy) is possible (Whaibeh et al., 2020; Wright and Caudill, 2020), many state that face-to-face contact is an essential part of the therapy (Connolly et al., 2020). Thus, the required reduction of face-to-face contacts might lead to a reduced number of patients (Humer et al., 2020; Probst et al., 2020) as some reservations against teletherapy have been shown in psychotherapists (Schuster et al., 2018) and the general population (Apolinário-Hagen et al., 2018). This situation might reinforce distress and job anxiety in psychotherapists, especially in those not used to provide teletherapy. Moreover, psychotherapists still providing psychotherapy face-to-face during COVID-19 might be especially stressed because of fear of becoming infected with COVID-19. Consequences of these examples might be increased mental burden of psychotherapists, and this distress may negatively impact process and outcome of psychotherapy (Salyers et al., 2017; La Verdière et al., 2018). The issue of preventing psychotherapists’ burnout is a central concern in this context. Research suggests that helpers who experience increased psychological distress are unable to respond optimally or to use their core skills (West and Shanafelt, 2007; Kitchingman et al., 2017).

Thus, exploring stress-level, job anxiety, and fear of COVID-19 infection in psychotherapists is essential to know if psychotherapists need to increase their mental hygiene during COVID-19. To the best of our knowledge whether and to what degree psychotherapists experience stress, job-related anxiety, and fear of infection in situations of exposing public health emergency, such as during the COVID-19 outbreak, have not been studied previously. Therefore, the current study aimed to investigate the stress-level, degree of job-related worries and fears of existence, as well as fear of COVID-19 infection in psychotherapists in the early weeks of the COVID-19 outbreak in Austria. Throughout the present study, job anxiety refers to generalized job-related worrying, as well as worrying about job security and the future.

In Austria, the first COVID-19 infections were reported on 25th of February 2020. To combat the rapid spread of the virus, a lockdown became obligatory on 16th of March 2020 (Bundesgesetzblatt für die Republik Österreich, 2020a,b,c). In general, entering public places was strictly prohibited. People were only permitted to leave their homes if they had a good reason for doing so, such as to meet necessary basic needs of daily life or to fulfill work responsibilities. In these exceptions, a minimum safe distance of 1 m (3 feet) had to be maintained between people. Certain areas in Austria were under quarantine at the time of the study and had even stronger restrictions.

In the present study, the following research questions (RQs) were addressed.

- RQ 1: How are stress-level, job-related worries and fears of existence, and fear of COVID-19 infection in psychotherapists in the early weeks of the COVID-19 outbreak? We hypothesized higher stress-level, as well as job-related worries and fears of existence than pre-pandemic scores from representative samples.
- RQ 2: Do stress-level as well as job-related worries and fears of existence differ between different groups of psychotherapists in the early weeks of the COVID-19 outbreak?
- RQ 2a: Are there differences between psychotherapists reporting that the psychotherapeutic work is their sole source of income and psychotherapists with other sources of income besides psychotherapy? We hypothesized that stress-level as well as job-related worries and fears of existence are higher if psychotherapy is the sole source of income.
- RQ 2b: Are there differences between psychotherapists who did not use teletherapy before the COVID-19 situation and psychotherapists who already used teletherapy before COVID-19? This RQ tested the hypotheses if psychotherapists used to teletherapy experience less stress-level as well as less job-related worries and fears of existence.
- RQ 2c: Are there differences between psychotherapists treating only face-to-face, treating face-to-face as well as *via* teletherapy, treating only *via* teletherapy, and psychotherapists not treating patients at all in the early weeks of the COVID-19 lockdown? We had no specific hypothesis here.
- RQ 2d: Are there differences between psychotherapists with more reductions (COVID-19 vs. months before) of total patients treated on average per week and psychotherapists with fewer reductions (COVID-19 vs. months before) of total patients treated on average per week? This RQ addressed the hypothesis whether more reductions of patients are associated with more stress-level as well as more job-related worries and fears of existence.
- RQ 3: Does fear of becoming infected with COVID-19 during face-to-face psychotherapy differ between different groups of psychotherapists?
- RQ 3a: Are there differences between psychotherapists treating patients face-to-face and psychotherapists not treating patients face-to-face? This RQ tested the hypothesis if psychotherapists still treating face-to-face have higher fear of COVID-19 infection.
- RQ 3b: For those psychotherapists treating patients face-to-face during COVID-19, does their ability to adhere to the protective measures against COVID-19 affect their fear of COVID-19 infection? This last RQ tested the hypothesis if psychotherapists being more able to adhere to the protective measures against COVID-19 have less fear of COVID-19 infection during face-to-face psychotherapy than psychotherapists being less able to adhere to the protective measures.

## Materials and Methods

### Study Design

In the current study, eligible participants included all licensed Austrian psychotherapists. In Austria, psychotherapy is an independent profession regulated by the Austrian law since 1990 (Psychotherapy Act, 361st Federal Act of June 7, 1990 on the Exercise of Psychotherapy). In brief, candidates have to complete a professional training comprising two stages (a general training followed by a specialist training) to qualify as a psychotherapist. All licensed psychotherapists in Austria are registered in the list of psychotherapists of the Austrian Federal Ministry of Social Affairs, Health, Care and Consumer Protection. In the current study, all psychotherapists who provided a valid e-mail address in this list (~6,000 psychotherapists of more than 9,000 licensed psychotherapists) were contacted by the first author in cooperation with the Austrian Federal Association for Psychotherapy (ÖBVP). Psychotherapists received a link to an online survey, which was open from 24th of March until 1st of April 2020. To start the survey, participants had to agree to the data protection declaration (electronic informed consent). No incentives were provided, and participation was voluntary. The survey followed the principles outlined in the Declaration of Helsinki, and the ethics committee of the Danube University Krems (Austria) approved the study.

### Measures

The Perceived Stress Scale with 10 items (PSS-10; Cohen et al., 1983) was used to measure the psychotherapists’ stress-level on a five-point response scale (0 = “never” and 4 = “very often”). The questions in this scale ask about feelings and thoughts during the last month, such as “How often have you been upset because of something that happened unexpectedly,” or “How often have you felt nervous and stressed.” The positively worded items of the PSS-10 (4, 5, 7, and 8) were reverse scored. The total score of the PSS-10 was obtained by summing up the items, so that higher scores indicate higher stress-level. In previous studies, Cronbach’s alpha of the PSS-10 was evaluated at >0.70, and test-retest reliability was >0.70 (see review by Lee, 2012). In our sample, Cronbach’s alpha was 0.83.

Job anxiety was measured with the 10 items of the “worrying and fear of existence” dimension of the Job Anxiety Scale (JAS; Linden et al., 2008). This dimension consists of the subscales “worrying” and “fears of existence” and has shown good internal consistency (Cronbach’s alpha: 0.88). The instruction was adapted, so that participants were asked to rate the statements in relation to the psychotherapeutic work in the current situation around COVID-19. Psychotherapists rated 10 statements that described situations, thoughts, and feelings which one can have experienced in connection with the workplace on a five-point response scale (0 = “strongly disagree” and 4 = “totally agree”). The “worrying” scale describes generalized worrying about minor matters concerning the workplace and the work itself, comprising of five items such as “Colleagues or family have already told me that I should not always worry that much about work.” The “fears of existence” scale focuses on worries about job security and the future, consisting of five questions like “A loss of my workplace is/would be existentially threatening.” The score for the worrying and fears of existence dimension was obtained by averaging the 10 items, with higher scores indicating more job-related worries and fears of existence. Values above the cut-off point of two points indicate high job-related worries and fears of existence (Muschalla et al., 2013). Cronbach’s alpha was 0.76 in our sample.

Psychotherapists were asked about their number of patients treated on average per week in the COVID-19 lockdown as well as (retrospectively) in the months before. These numbers were given for face-to-face psychotherapies, for psychotherapy *via* telephone, and for psychotherapy *via* internet. For psychotherapists not treating during/before COVID-19, these numbers were set to 0. Using these numbers, reductions of total (face-to-face, telephone, and internet) number of patients treated on average per week during COVID-19 vs. in the months before were calculated (number in the months before COVID-19 was subtracted from the number during COVID-19, i.e., during COVID-19 – before COVID-19, so that more negative values indicate more reductions). As reported in another paper (Probst et al., 2020), the reductions of patients treated on average per week was statistically significant [*M* = 3.92 (*SD* = 11.04), *p* < 0.001].

Psychotherapists were asked whether psychotherapy is their sole source of income or whether they have additional sources of income.

Psychotherapists were asked to rate their fear to become infected with COVID-19 during psychotherapy in which they are face-to-face with patients on a sliding scale ranging from 0 (“not at all”) to 100 (“extreme”).

Psychotherapists treating patients face-to-face during the COVID-19 lockdown rated for each of the five protective measures against COVID-19 how well they can adhere to the protective measure during face-to-face psychotherapy on a four-point response scale (1 = “cannot adhere to the measure at all” and 4 = “can completely adhere to the measure”). The following five protective measures were suggested by the government (Austrian Federal Ministry of Social Affairs, Health, Care and Consumer Protection, 2020): (1) wash your hands frequently! Regularly and thoroughly wash your hands with soap or clean them with a disinfectant. (2) Maintain social distancing! Maintain at least 1 m (3 feet) distance between yourself and all other persons who are coughing or sneezing. (3) Do not touch eyes, nose, and mouth! Hands can pick up viruses and transfer the virus to your face! (4) Practice respiratory hygiene! Cover your mouth and nose with your bent elbow or tissue when you cough or sneeze and dispose of the used tissue immediately. (5) If signs and symptoms occur, do not leave your home and contact health care professionals or emergency services by phone.

### Statistical Analyses

Statistical analyses were performed with SPSS25 (IBM Analytics).

Descriptive statistics were calculated to characterize participants and address RQ 1. The comparison of the PSS-10 with a norm sample was conducted using a *t*-test. For the job-related worries and fears of existence dimension of the JAS, we compared the average score against the cut-off of two points indicating high job-related worries and fears of existence.

To address RQ 2a,b and RQ 3a, independent *t*-tests were used to compare two groups of psychotherapists in each RQ. For RQ 2c, univariate ANOVAs were performed to investigate four groups of psychotherapists.

To address RQ 2d and RQ 3b, Pearson’s correlation analysis was performed.

We report effect sizes using Hedge’s *g* with 95% CIs. All statistical tests for significance were conducted two-tailed with an alpha level of 0.05.

## Results

### Participant Characteristics

In total, 1,547 psychotherapists participated. Their mean age was 51.67 (*SD* = 9.69) years, and 75.7% of them were female. A comparison of the distribution of their psychotherapeutic orientations with the distribution of therapeutic orientations in the official Austrian list of psychotherapists (March 2020) showed that the humanistic orientation was overrepresented in the survey (% in the study sample vs. % in the Austrian list of psychotherapists): psychodynamic 20.9 vs. 25.9%, humanistic 46.3 vs. 37.8%, systemic 22.0 vs. 24.3%, and behavioral 9.8 vs. 12.0% (not specified for 1% of the survey sample). The average year in profession (indicated as the time since psychotherapists were registered in the Austrian list of psychotherapists in March 2020) was 11.19 (*SD* = 9.20) years. Of the participating psychotherapist, 781 (50.5%) were treating only adults, 14 (0.9%) only children and adolescents, and 752 (48.6%) adults as well as children and adolescents. In total, 1,234 psychotherapists (79.8%) were self-employed practitioners, 32 (2.1%) were regularly employed, and 281 (18.2%) worked self-employed as well as regularly employed.

#### Results for RQ 1

The average stress-level of the participating psychotherapists on the PSS-10 was *M* = 13.27 (*SD* = 5.85). Compared to the stress-level of employed persons in a representative German sample (*M* = 12.32, *SD* = 6.30; Klein et al., 2016), the stress-level of the psychotherapists was higher, *p* < 0.001, but the effect size was very small, Hedge’s *g* = 0.16, 95% CI = 0.08, 0.23.

On average, psychotherapists scored *M* = 0.71 (*SD* = 0.50) on the “worrying and fears of existence” dimension of the JAS, thus scoring below 2.0, the threshold differentiating between low and high job-related anxiety in a nonclinical employees sample (Muschalla et al., 2013).

The average fear to become infected with COVID-19 during face-to-face psychotherapy was *M* = 37.51 (SD = 28.34).

#### Results for RQ 2a

Compared to psychotherapists with additional sources of income (*n* = 707), psychotherapists whose income relied solely on psychotherapy (*n* = 840) reported significantly higher stress-levels, *T*(1,530.60) = 2.333, *p* = 0.020, Hedge’s *g* = 0.12, 95% CI = 0.02, 0.22, and higher job-related worrying and fears of existence, *T*(1,543.25) = 7.07, *p* < 0.001, Hedge’s *g* = 0.36, 95% CI = 0.26, 0.46. Means and SDs are shown in Table 1.

**Table 1:** Comparison of perceived stress and job-related worrying and fears of existence in relation to income sources of psychotherapists.

Outcome	Additional income sources (*n* = 707)		Psychotherapy as sole income (*n* = 840)		*T*	*p*	Hedge’s *g*
	*M*	*SD*	*M*	*SD*			
Perceived stress	12.89	5.60	13.58	6.04	2.333	0.020	0.12
Job-related worrying and fears of existence	0.612	0.458	0.788	0.526	7.070	<0.001	0.36

Perceived Stress was measured with the 10-items version of the Perceived Stress Scale (PSS-10; Cohen et al., 1983). Job-related worrying and fears of existence were measured with the 10 items “worrying and fears of existence” dimension of the Job Anxiety Scale (JAS; Linden et al., 2008). Mean parameter values for each of the analyses are shown for the psychotherapists with psychotherapy as sole income (*n* = 840) and the psychotherapists with additional sources of income (*n* = 707), as well as the results of the two-tailed *t*-tests (assuming unequal variance) comparing the parameter estimates between the two groups of psychotherapists.

#### Results for RQ 2b

Compared to psychotherapists who already used telephone or internet for psychotherapy in the months before COVID-19 (*n* = 316), psychotherapists who did not use telephone or internet for psychotherapy in the months before COVID-19 (*n* = 1,231) reported no differences regarding perceived stress, *T*(1,545) = 1.246, *p* = 0.213, Hedge’s *g* = 0.079, 95% CI = −0.05, 0.20, and job-related worrying and fears of existence, *T*(1,545) = 0.397, *p* = 0.692, Hedge’s *g* = 0.024, 95% CI = −0.10, 0.15. Table 2 shows the means and SDs.

**Table 2:** Comparison of perceived stress and jobs-related worrying and fears of existence in relation to experience with teletherapy (telephone or internet) in the months before COVID-19.

Outcome	Teletherapy before COVID-19 (*n* = 316)		No teletherapy before COVID-19 (*n* = 1,231)		*T*	*p*	Hedge’s *g*
	*M*	*SD*	*M*	*SD*			
Perceived stress	12.90	5.90	13.36	5.83	1.246	0.213	0.079
Job-related worrying and fears of existence	0.698	0.521	0.710	0.499	0.397	0.692	0.024

Perceived Stress was measured with the 10-items version of the PSS-10 (Cohen et al., 1983). Job-related worrying and fears of existence were measured with the 10 items “worrying and fears of existence” dimension of the JAS (Linden et al., 2008). Mean parameter values for each of the analyses are shown for the psychotherapists who already used teletherapy (telephone or internet) in the months before COVID-19 (*n* = 316) and the psychotherapists who did not use telephone or internet for psychotherapy in the months before COVID-19 (*n* = 1,231), as well as the results of the two-tailed *t*-tests (assuming equal variance) comparing the parameter estimates between the two groups of psychotherapists.

#### Results for RQ 2c

Between psychotherapists treating only face-to-face (*n* = 31), face-to-face as well as *via* teletherapy (telephone or internet, *n* = 618), only *via* teletherapy (telephone or internet, *n* = 793), or not at all (*n* = 105) in the early weeks of the COVID-19 lockdown, stress-levels, *F*(3, 1,543) = 1.462, *p* = 0.223, and job-related worries and fears of existence, *F*(3, 1,543) = 0.304, *p* = 0.823, did not differ. Of the 105 psychotherapists treating not at all 71 reported that they treated patients in the months before COVID-19, whereas 34 reported that they did not. Means and SDs are shown in Table 3.

**Table 3:** Comparison of perceived stress and job-related worrying and fears of existence in relation to the practice of psychotherapy (only in face-to-face, face-to-face and *via* teletherapy, only *via* teletherapy, and not at all) in the early weeks of the COVID-19 lockdown.

Outcome and group	*M*	*SD*	*F*(3,1,543)	*p*
Perceived stress			1.462	0.223
Psychotherapy only face-to-face	13.16	5.92		
Psychotherapy face-to-face and *via* teletherapy	13.13	5.91		
Psychotherapy only *via* teletherapy	13.23	5.80		
No Psychotherapy at all	14.41	5.76		
Job-related worrying and fears of existence			0.304	0.823
Psychotherapy only face-to-face	0.694	0.555		
Psychotherapy face-to-face and *via* teletherapy	0.718	0.497		
Psychotherapy only *via* teletherapy	0.704	0.516		
No Psychotherapy at all	0.671	0.435		

Perceived Stress was measured with the 10-items version of the PSS-10 (Cohen et al., 1983). Job-related worrying and fears of existence were measured with the 10 items “worrying and fears of existence” dimension of the JAS (Linden et al., 2008). Mean parameter values for each of the analyses are shown for the psychotherapists who treated only face-to-face (*n* = 31), face-to-face and *via* teletherapy (telephone or internet; *n* = 618), only *via* teletherapy (telephone or internet; *n* = 793), or not at all (*n* = 105) in the early weeks of the COVID-19 lockdown.

#### Results for RQ 2d

Psychotherapists with more reductions in the total (face-to-face + telephone + internet) number of patients treated on average per week in COVID-19 as compared to the months before experienced comparable stress-level, *r* = −0.006, *p* = 0.818, as well as comparable job-related worries and fears of existence, *r* = −0.011, *p* = 0.660, as psychotherapists with less reductions in the total number of patients treated on average per week.

#### Results for RQ 3a

Psychotherapists who conducted no face-to-face psychotherapy in the early weeks of the COVID-19 lockdown (*n* = 898) reported higher fear of infection (*M* = 43.48, *SD* = 29.65) compared to the 649 psychotherapists who still conducted face-to-face psychotherapy during the COVID-19 lockdown (*M* = 29.26, *SD* = 24.13), *T*(1,523.39) = 10.383, *p* < 0.001, Hedge’s *g* = 0.52, 95% CI = 0.42, 0.62.

#### Results for RQ 3b

Table 4 shows the means and SDs regarding how well psychotherapists could adhere to the five protective measures against COVID-19 during face-to-face psychotherapy in the early weeks of the COVID-19 lockdown. In addition, the correlation coefficients for associations between the psychotherapists’ ability to adhere to the protective measures and fear of COVID-19 infection during face-to-face psychotherapy are given in Table 4.

**Table 4:** Ability to adhere to the protective measures against COVID-19 as proposed by the Austrian government during face-to-face psychotherapy in the early weeks of the COVID-19 lockdown and correlations with fear of COVID-19 infection.

Protective measure against COVID-19	*M*(SD)	Correlation (*r*) with fear of COVID-19 infection
Wash your hands frequently! Regularly and thoroughly wash your hands with soap or clean them with a disinfectant	3.89(0.43)	−0.20^**^
Maintain social distancing! Maintain at least 1 m (3 feet) distance between yourself and all other persons who are coughing or sneezing	3.81(0.53)	−0.21^**^
Do not touch eyes, nose and mouth! Hands can pick up viruses and transfer the virus to your face!	3.24(0.82)	−0.13^**^
Practice respiratory hygiene! Cover your mouth and nose with your bent elbow or tissue when you cough or sneeze and dispose of the used tissue immediately.	3.82(0.52)	−0.22^**^
If signs and symptoms occur, do not leave your home and contact health care professionals or emergency services by phone.	3.88(0.47)	−0.12^**^

Fear to become infected with COVID-19 during face-to-face psychotherapy was rated on a sliding scale ranging from 0 (“not at all”) to 100 (“extreme”). Ability to adhere to the five protective measures against COVID-19 during face-to-face psychotherapy was rated on a four-point response scale (1 = “cannot adhere to the measure at all” and 4 = “can completely adhere to the measure”). Results refer to the *n* = 649 psychotherapists treating face-to-face during the COVID-19 lockdown in Austria; *r*, Pearson’s correlation coefficient.

** *p* < 0.01 two-tailed.

The correlation coefficients between ability to adhere to the protective measures and fear of COVID-19 infection were all negative and statistically significant (all values of *p* < 0.01). This means that psychotherapists treating face-to-face during the COVID-19 lockdown had significantly less fear of COVID-19 infection when they were more able to adhere to the protective measures against COVID-19.

## Discussion

This survey explored stress-level, job-related worries and fears of existence, and fear of COVID-19 infection during face-to-face psychotherapy in psychotherapists in Austria. Stress-level was higher than scores of a German-speaking norm sample. Job-related worries and fears of existence were below the cut-off that defines high job-related anxiety. These results confirm the hypothesis that stress-level was elevated, but reject the one that job-related worries and fears of existence were high.

Stress-level and job-related worries and fears of existence were significantly higher in psychotherapists who had no other sources of income besides psychotherapy. This confirms our hypothesis. Since mental well-being of psychotherapists represents a key determinant of their ability to deliver high-quality psychological health care (Salyers et al., 2017; La Verdière et al., 2018), this illustrates the need to reduce existential stressors due to economic uncertainty, especially for psychotherapists whose income relies solely on psychotherapy. Besides professional policy, stress-management interventions for health care professionals might be further options for psychotherapists who derive all their income from psychotherapy (Ruotsalainen et al., 2015).

Stress-level and job-related worries and fears of existence were not lower for psychotherapists who practiced psychotherapy *via* telephone or internet already before COVID-19. This result contrasts with our hypothesis assuming that those psychotherapists already used to teletherapy experience less stress-level, as well as job-related worries and fears of existence during COVID-19. Maybe switching to telephone or internet to provide psychotherapy was easy for those psychotherapists who did not use these formats for psychotherapy before COVID-19. It has also been reported that in the context of the forced transition toward teletherapy because of the COVID-19 pandemic, the majority of 145 surveyed psychotherapists from North America and Europe developed a positive attitude toward teletherapy (Békés and Aafjes-van Doorn, 2020). Therefore, it is possible that psychotherapists without previous teletherapy experience felt more at ease using teletherapy after they gained first experiences. Also previous studies showed that therapists reported that they were pleasantly surprised by the functionality and ease of use of videoconferencing upon using teletherapy (Connolly et al., 2020). The context of this forced transition to teletherapy because of the COVID-19 pandemic might have further increased the psychotherapists’ motivation to use remote psychotherapy in order to be able to continue the sessions with all or most of their patients.

Stress-levels as well as job-related worries and fears of existence did not differ between psychotherapists treating only face-to-face, face-to-face as well as *via* teletherapy, only *via* teletherapy, or not at all. One explanation why psychotherapists treating not at all during COVID-19 did not differ from the other groups regarding stress and job anxiety might be that they did not depend financially on psychotherapy. Indeed, about one-third (34 out of 105) of the psychotherapists providing no psychotherapy at all during COVID-19 did not treat patients in the months before COVID-19, either. Thus, they could afford to quit practicing during the lockdown without additional stress and job-related worries. However, one has also to consider that both groups of psychotherapists were rather small (*n* = 31 psychotherapists practicing only face-to-face and *n* = 105 psychotherapists practicing not at all), which limits the overall significance of the current findings.

Details on the number of patients treated with respect to treatment format have been published recently (Probst et al., 2020). In brief, the total number of patients treated on average per week decreased from *M* = 14.04 (*SD* = 11.32) in the months before the COVID-19 lockdown to *M* = 10.12 (*SD* = 9.05) in the early weeks of the COVID-19 lockdown (*p* < 0.001). Reductions in total number of patients treated on average per week in COVID-19 as compared to the months before affected neither stress-level nor job-related worries and fears of existence. This result is in contrast to our hypothesis that more reductions in patients treated are associated with more stress-levels as well as more job-related worries. One explanation for this could be that, in the early weeks of the COVID-19 lockdown, psychotherapists were hoping that the lockdown will soon be over and that they will be able to treat their usual number of patients by face-to-face psychotherapy soon again. The longer the lockdown, the higher the correlations (between reduced number of patients on the one side and stress-level or job-related worries or fears of existence on the other side) might be.

Psychotherapists still practicing face-to-face during the COVID-19 lockdown had lower fear of COVID-19 infection during face-to-face psychotherapy than psychotherapists not practicing face-to-face during COVID-19. This result rejects our hypothesis that fear of COVID-19 infection during face-to-face psychotherapy is higher in psychotherapists still treating patients face-to-face during COVID-19. An explanation for this result might be that fear of COVID-19 infection might be a reason for some psychotherapists to stop treating face-to-face. Furthermore, it might be that those psychotherapists who have limited practice space, stopped treating face-to-face as they would not have been able to keep an appropriate safety distance. This is further supported by the negative correlation between the adherence to the protective measure of social distancing and the fear of COVID-19 infection in psychotherapists treating face-to-face during the lockdown. Similarly, also the ability to adhere to the other four protective measures against COVID-19 of the Austrian government was associated with lower fear of COVID-19 infection for psychotherapists treating face-to-face.

It should be kept in mind that most effect sizes for the significant results were small. The results refer to the early weeks of the COVID-19 situation in Austria (first COVID-19 infections were reported on 25th of February 2020, measures of the government became obligatory on 16th of March 2020, and the survey was open from 24th of March to 1st of April 2020). Stress-levels and job-related anxiety might change dynamically either positively or negatively depending on the durations and intensity of the restrictions.

There are a number of limitations in this study. The major limitation is the cross-sectional design, so that we cannot say whether the psychotherapists’ stress-level or job-related worries and fears of existence changed during COVID-19 as compared to the time before. A further limitation is that the fear to become infected was operationalized by a single item measure. Meanwhile, a validated scale to assess the fear of COVID-19 became available (Ahorsu et al., 2020), which should be considered in future studies. In addition, only psychotherapists’ self-ratings on number of patients treated on average per week could be analyzed and not health insurance data. Due to the cross-sectional design, there might be a recall bias regarding the number of patients treated on average per week in the months before COVID-19. Moreover, stress-level was operationalized only with self-reports and not complemented by more objectively quantifiable physiological measurements, such as cortisol analyses (Dickerson and Kemeny, 2004). Such analyses are not easily possible in online surveys, and lab studies would be necessary. Another shortcoming is the online conduction of the survey, which might have caused some respondent bias, such as higher psychotherapists’ participation with higher preference for new technologies, which might have contributed to the finding that experience with teletherapy did not affect stress-level and job-related anxiety. Carrying out the survey online may also have introduced some selection bias toward fewer elder psychotherapists’ participation (Bethlehem, 2010). Although the sample largely reassembled the psychodynamic, behavioral, and systemic population of Austrian therapists (deviation range from −5.0 to −2.2% units), therapists with a humanistic orientation were overrepresented (deviation range of 8.5% units), which further limits the generalizability of the findings to the population of Austria’s psychotherapists. Since the study was conducted in Austria, results may only be applicable to countries with similar mental health care systems (for example, psychotherapy – but not counseling – *via* internet is rejected by the official Internet guideline for psychotherapists in Austria at the time of the study; however, health insurances started to cover the costs for psychotherapy *via* telephone or internet during COVID-19) and comparisons with countries, which already implemented e-health solutions in routine psychotherapy would be interesting.

## Conclusion

Overall, psychotherapists need to meet the challenges inherent in balancing stressors, especially in situations of increased mental, emotional, and economic challenges, such as during COVID-19, to ensure optimal psychotherapeutic services. This study suggests that mental hygiene is important for psychotherapists to manage stress and job-related anxiety during COVID-19. The finding that mainly being financially dependent on psychotherapy was associated with higher stress-level and job anxiety is important in regard to professional policy. This might also have an effect on therapeutic process, as increased mental burden of psychotherapists and distress may negatively affect process and outcome of psychotherapy. Therefore, results suggest that especially psychotherapists whose income relies on psychotherapy need to increase their mental hygiene during COVID-19.

## Data Availability Statement

The raw data supporting the conclusions of this article will be made available by the authors, without undue reservation.

## Ethics Statement

Ethical review and approval was not required for the study on human participants in accordance with the local legislation and institutional requirements. The patients/participants provided their written informed consent to participate in this study.

## Author Contributions

TP, PS, and CP: conceptualization. TP: methodology, formal analysis, investigation, and data curation. TP and EH: writing – original draft preparation. CP and PS: writing – review and editing. All authors have read and agreed to the published version of the manuscript.

### Conflict of Interest

PS has no conflict of interest related to the study, but, in his function as president of the Austrian Federal Association for Psychotherapy, he is interested in representing the psychotherapists well.

The remaining authors declare that the research was conducted in the absence of any commercial or financial relationships that could be construed as a potential conflict of interest.
